# Experiencing fertility preservation in adolescence – a qualitative interview study indicating gender disparities in AYAs diagnosed with cancer

**DOI:** 10.3389/fonc.2025.1515952

**Published:** 2025-02-19

**Authors:** Kenny A. Rodriguez-Wallberg, Hanna Nilsson, Maria Folmerz, Erica Lundqvist, Lisa Granberg, Gabriela Armuand

**Affiliations:** ^1^ Department of Reproductive Medicine, Division of Gynecology and Reproduction, Karolinska University Hospital, Stockholm, Sweden; ^2^ Department of Oncology-Pathology, Karolinska Institutet, Stockholm, Sweden; ^3^ Department of Obstetrics and Gynecology, Örebro University, Örebro, Sweden; ^4^ Department of Women’s Health Care, Karlstad Central Hospital, Karlstad, Sweden; ^5^ School of Health and Social Studies, Dalarna University, Falun, Sweden

**Keywords:** adolescents, young adults, AYAs, cancer, cancer treatment, fertility preservation, infertility, neoplasm

## Abstract

**Introduction:**

Fertility counselling on options for fertility preservation is increasingly implemented for children and adolescents at time of cancer diagnosis. Sperm cryopreservation has been standard of care for male patients during several decades and the procedure is not expected to delay the onset of cancer treatment. However, oocyte cryopreservation in female adolescents remains controversial, the reasons include the need of ovarian stimulation, gynecological exams and interventions, in all a potentially distressing experience for patients without previous experience of this type of examination or without previous sexual debut. With this study we wished to investigate how adolescent cancer patients experience fertility preservation procedures aiming at semen banking or oocyte cryopreservation.

**Methods:**

Adolescent patients diagnosed with cancer that underwent fertility preservation at the Reproductive Medicine Clinic of Karolinska University Hospital were invited to participate in the study. Inclusion required the ability to communicate in Swedish or English. Exclusion criteria were current age under 15 at time of the interview and ongoing cancer treatment. The study had a qualitative study design and phenomenological approach with semi-structured individual face-to-face interviews. Ten interviews with six female and four male study participants were conducted between June and August 2023.

**Results:**

The analysis resulted in three identified main themes: Communication about the risk of infertility and the fertility preservation, Freezing gametes - the process and healthcare encounters, and The decision to preserve gametes for one’s own sake. Gender specific gaps in communication about fertility risks and fertility preservation procedures were found, with young females expressing a wish for improved communication and reporting experiences of discomfort during the procedures needed for oocyte cryopreservation, whereas young men were generally satisfied with their experience. Limitations include a risk of responder bias since not all patients who were contacted agreed to interview.

**Discussion:**

Although gender disparities were identified in this study, fertility preservation was perceived as a positive experience and mitigated fertility-related distress in both male and female adolescent patients. Our study adds to the scarce literature on adolescents of both sexes undergoing fertility preservation and underscores the importance of specialized communication in fertility counselling and treatment of AYAs diagnosed with cancer.

## 1 Introduction

Nowadays most adolescents and young adults (AYAs) suffering from cancer have a high likelihood of becoming long-term survivors. Data indicate that a large percentage of AYA cancer survivors display a strong wish for biological parenthood (1). However, cancer treatment may negatively affect the fertility potential of these individuals, leading to reproductive concerns and fertility-related distress (2–6). The concerns are well-founded, as a cancer diagnosis before the age of 40 significantly reduces both the likelihood to achieve pregnancy and to have future biologically-related children (7–9). In their updated evidence-based guidelines, published 2018, the American Society of Clinical Oncology (ASCO) indicated the need for fertility counselling and referral for fertility preservation (FP), if possible before initiation of cancer treatment (10).

Sperm cryopreservation has been standard-of-care for male adolescents for several decades, and it is feasible once spermarche has been achieved and the testis volume has reached 8 ml (11). The procedure is not expected to delay the onset of cancer treatment. Oocyte cryopreservation, on the other hand, was only recognized as a clinical treatment a decade ago (10), and although it can offer an option to preserve mature oocytes for adult women and post-pubescent girls, the procedure requires continued healthcare interventions, transvaginal exams and ovarian puncture for follicle aspiration, as well as time for ovarian stimulation with gonadotropins. If the stimulation protocol can be applied with random start, the time to egg retrieval averages about two weeks. Studies on patients with breast cancer undergoing FP at adult age, have not shown any increase in disease-specific mortality or relapse among the women undergoing FP (12). However, oocyte cryopreservation in female adolescents remains controversial due to several factors including limited experience of applying protocols for ovarian stimulation on an immature ovary, possibly causing an inappropriate ovarian response (13–15), the necessity to rapidly start a treatment using daily injections, ultrasound, and blood work (15) and the limited experience among young patients of gynecological examinations or transvaginal insertions (16). As the procedures surrounding sperm cryopreservation are less demanding physically when compared to oocyte cryopreservation, it is not surprising that utilization of FP is higher among young men than women (17, 18). However, gender disparities have been reported not only in the performance of FP in young adults with cancer, but also in their counselling, indicating that young men are more likely to be informed on potential infertility outcomes following cancer treatment, and more often offered methods for FP, than young women facing similarly gonadotoxic treatments (19).

For pre-pubertal patients, additional FP methods include the retrieval of ovarian and testicular tissues for cryopreservation. These options have been initiated across Europe since the early 2000’s within Ethics Review Board approved research protocols (20, 21). The long-term experience with these methods applied to prepubertal children has been reported from a prospective cohort, supporting the feasibility and the safety of these approaches (22, 23). While results on tissue transplantation to recover fertility potential are still limited among patients that were prepubertal at tissue cryopreservation, ovarian tissue transplantation has demonstrated efficacy and robustness in adult women (24). However, there is a need of continued research to develop methods for *in vitro* gametogenesis for the cases where transplantation of the gonadal tissue is precluded, which is highlighted in the current international guidelines for AYAs with cancer (10, 25–27).

The international guidelines for FP in children and teenagers (25, 26), are developed to ensure that healthcare providers are familiar with the available methods and can provide equal access to FP counselling for children and teenagers with cancer. With this study we wished to investigate how adolescents treated for cancer experienced fertility counselling and fertility preservation procedures at a reference center specialized on FP within the public Swedish healthcare system.

## 2 Materials and methods

The study had a qualitative design and phenomenological approach with semi-structured individual face-to-face interviews. Study participants were individuals diagnosed with cancer at adolescence and referred to the Reproductive Medicine Clinic of Karolinska University Hospital for fertility preservation. Referrals of patients with oncologic indications are accepted for FP without delay when a cancer treatment with potential negative on fertility is planned. Additionally, survivors of childhood cancer that were not referred at time of diagnosis may be referred several years after completion of treatment, at transfer to adult healthcare. The reproductive Medicine Clinic of Karolinska University Hospital is the largest in Sweden and have been counselling and treating female and male adolescent patients for over twenty years (28, 29).

**Figure 1:**
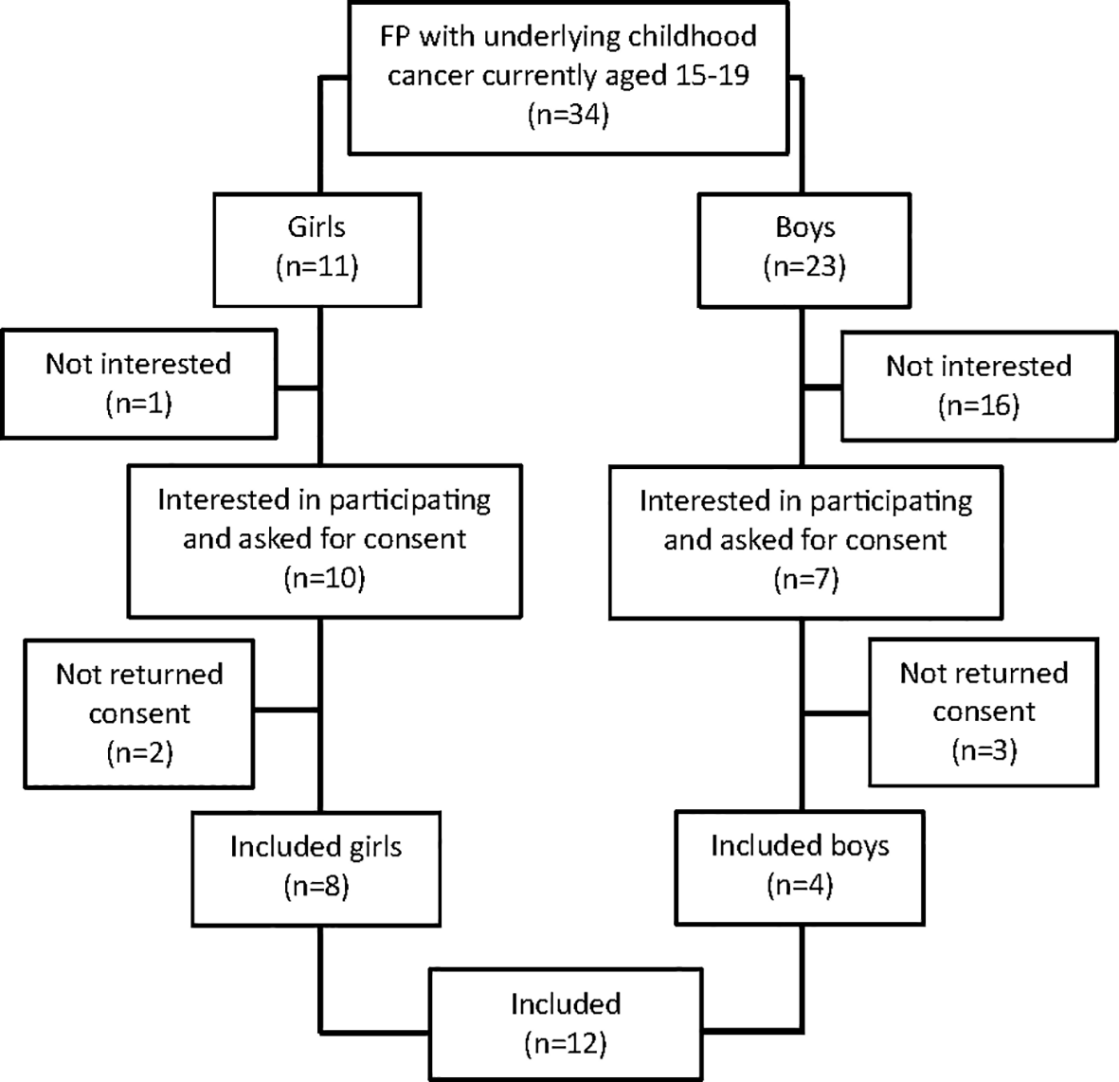
Study flow chart. FP, Fertility Preservation.

At time of counselling, patients had given informed consent to participate in a prospective follow-up of clinical outcomes and permission to contact them for future interview studies. For this study, individuals were approached after having accessed FP indicated by a cancer diagnosis during childhood or adolescence. Exclusion criteria were age under 15 at the time of the interview, having an ongoing cancer treatment or being unable to communicate in Swedish or English. Individuals were contacted by phone (n =34) and a letter with study information together with a study specific consent form was sent to those that expressed interest to participate in the study (n=17), among them twelve signed the consent but two individuals were not available thereafter after repeated contact attempts, thus ten individuals were interviewed (**Figure 1**). The interviews were conducted on the online platform Zoom by GA between June and August 2023. The interviews were conducted in Swedish and, in one case, in English, using open-ended questions from a semi-structured interview guide developed by GA. GA had not been previously involved in the individuals’ healthcare.

The interviews commenced with the overarching question: *How was your experience of freezing eggs/sperm?* Subsequently, the interviews followed the participants’ narratives but were guided, when necessary, to cover experiences of the following areas: *Risk of infertility and thoughts about the future*, *Fertility preservation procedures*, and *Healthcare encounters*. Follow-up questions were posed as needed to deepen or clarify the informant’s narrative, such as *What did you mean when you said…?* or *Could you tell me more about…?* After each interview, field notes were documented, capturing impressions of the interview situation and the elements that were particularly prominent in the participant’s narrative. The interviews lasted between 18 and 47 minutes, with an average duration of 30 minutes and were digitally recorded and transcribed verbatim. The study follows the Standards for Reporting Qualitative Research (SRQR) and is reported according to the SRQR Checklist (30).

Data was analyzed by content analysis (31) using an inductive approach. Through open coding, units of meaning associated with the study objective were identified and condensed into codes that mirrored their content. Codes conveying similar content were subsequently grouped into categories, and these categories were further organized hierarchically into main categories and sub-categories. Quotations provided to support the content (30), are presented with the interviewer’s questions, clarifications, and omissions (indicated by three dots) denoted within squared brackets (**Tables 1**, **2**).

**Table 1:** Example of the open coding process from units of meaning to codes.

	Condensation	Codes
It was a bit scary. Especially when you had to wear these kinds of surgical clothes, and it was very clinical. But I understand that it has to be that way. But I thought it was nerve-wracking. At least in the beginning.	Scary, clinical, and nerve-wracking at first during the egg retrieval.	Scary and nerve-wracking to undergo egg retrieval
They didn't really tell me that there would be any large risk [for infertility], just whether I wanted to freeze them or not, since I only did one round [of chemotherapy]. But I wanted to. I just thought; why not just do it and have it there in case something happens.	Freezing sperm despite low risk in case something happens.	Proactive sperm freezing despite low risk
You start thinking a lot about it [the fertility], or at least I did, especially at that age when you hit puberty and you want to know everything, like what's happening in your body [ … ]. Then you want to keep track of it I think [the risk of infertility], as early as possible.	Awareness of bodily changes during puberty and desire to know infertility risks early on.	Early fertility awareness during puberty wanted
She [the midwife] was really nice. [ … ] She tried to explain everything [about the injections] in an undramatic way, and she was really nice.	The midwife was nice and explained about the injections in an undramatic way.	Compassionate and clear communication about medical procedures

**Table 2:** Example of the open coding process from codes to main categories.

Codes	Sub-category	Main category
Early fertility awareness during puberty wanted	To become aware of the risk of infertility	Communication about the risk of infertility and the fertility preservation
Overwhelmed by information about cancer and risk of infertility and at the same time		
Compassionate and clear communication about medical procedures	Information satisfaction	
Positive to have visual information about the process		
Lack of information led to a negative experience of vaginal ultrasound	Insufficient information	
Vague information about the sperm banking process		
Uncomfortable with the gynecological examination but got used to it	Preparatory examinations and treatment	Freezing gametes - the process and healthcare encounters
Giving myself the injection was creepy		
Scary and nerve-wracking to undergo egg retrieval	Oocyte retrieval and sperm donation	
The process [of sperm banking] was nothing to worry about		
The staff managed the stressful situation with calmness and support	Healthcare interactions throughout the process	
Lack of time hampered modified information to the individual		
Wouldn't have undergone [oocyte cryopreservation] if the doctor hadn't recommended it.	The complexity of deciding to cryopreserve gametes	The decision to preserve gametes for one's own sake
Went to several appointments to discuss options [for fertility preservation]		
Proactive sperm freezing despite low risk	Better safe than sorry	
I might have regretted it and obsessed over it if I hadn't gone through with the treatment.		

The study was approved by the Swedish Ethical Review Authority (Dnr 2011/1758-31/2, amendment Dnr 2014/286-32, 2018/275-32 and 2022-05969-02).

## 3 Results

In all, ten individuals were interviewed, six female and four males. During one interview, a legal guardian participated, providing support for the informant. There was no difference in the average age between genders at the time of the interview, nor in the age at FP; however, the average age at the time of diagnosis was lower among females than among males. The diagnoses included Hodgkin’s lymphoma (n=4), Rhabdomyosarcoma (n=1), Testicular cancer (n=1), Germinoma (n=1), Ovarian cancer (n=1), Soft tissue sarcoma in the uterus/bladder (n=1), and Colon cancer (n=1). Additional socio-demographic data are shown in **Table 3**. All male participants were post pubertal at diagnosis and were referred from their pediatric oncologists and all had cryopreserved sperm as an acute measure before initiating cancer treatment. Two females, post pubertal at diagnosis, had received counselling at time of cancer treatment and undergone emergency FP, The remaining four girls were prepubertal at diagnosis and were not referred to the fertility clinic for counselling at diagnosis, but received a referral for reproductive counselling when being transferred to adult healthcare several years after completion of their cancer treatment. All female participants chose to proceed with oocyte cryopreservation. All study participants cryopreserved gametes successfully.

**Table 3:** Socio-demographics (n=10).

Variables	Females (n=6)	Males (n=4)
	Mean (min-max)	Mean (min-max)
Age at interview	19.3 (17–21)	19.5 (18–21)
Age at FP	18,3 (17-19)	18,3 (17-19)
Age at diagnosis	11.3 (5–19)	18.3 (17–19)
	n (%)	n (%)
Place of residence		
Large city	5 (83.3)	3 (75.0)
Medium-sized city	1 (16.7)	0 (0.0)
Village/Rural area	0 (0.0)	1 (25.0)
Occupation		
Compulsory school **	1 (16.7)	0 (0.0)
Secondary education***	3 (50.0)	2 (50.0)
University	1 (16.7)	2 (50.0)
Adult education	1 (16.7)	0 (0.0)
Employment	2 (33.3)	1 (25.0)

** 10 years from age 6 to 16.

*** Two to four years of education after compulsory school.

During the analysis, three main categories were identified: *Communication about the risk of infertility and the fertility preservation*, *Freezing gametes - the process and healthcare encounters*, and *The decision to preserve gametes for one’s own sake*. These three main categories have eight subcategories (**Table 4**).

**Table 4:** Overview of theme, main categories, and sub-categories.

Communication about the risk of infertility and the fertility preservation	Freezing gametes - the process and healthcare encounters	The decision to preserve gametes for one’s own sake
To become aware of the risk of infertility	Preparatory examinations and treatment	The complexity of deciding to cryopreserve gametes
Information satisfaction	Oocyte retrieval and sperm donation	Better safe than sorry
Insufficient Information	Healthcare interactions throughout the process	

### 3.1 Communication about the risk of infertility and undergoing fertility preservation

#### 3.1.1 To become aware of the risk of infertility

The received information about the risk of infertility was perceived as direct and straightforward, and the information seen as needed and positive. Fertility preservation thus became a natural step in the treatment plan for their cancer diagnosis. However, when the information was given in conjunction with informing about the cancer diagnosis, it was perceived as overwhelming and challenging to absorb. The study participants indicated that it would have been preferable if the FP information had been provided separately, after the shock of the cancer diagnosis had subsided. For some of the young individuals, their attending physicians did not provide information about the risk of infertility. Instead, the question was initiated by a close relative during the medical appointment with the pediatric oncologist. The study participants indicated that they would have preferred if the information about the risk of infertility had been initiated by the pediatric oncologist.

It wasn’t a doctor who said it. [ … ] It was [a relative] who was present at a meeting and said [ … ] that she had heard that some people become sterile or that there’s a risk. So, she asked about it, and they [said]: ‘Yes, you can freeze it if you want to’.

Male, age 22

Some AYAs who did not receive information on infertility risks at time of treatment, expressed frustration over the delay and how information was conveyed. The information about the potential treatment impact on fertility only reached them during their late teenage years, at the time of transfer to adult healthcare. They perceived that the information on infertility risk was provided only in direct connection with the offer to cryopreserve gametes. This lack of early information resulted in feelings of anger and sadness. They wished they had received this information earlier in life, preferably at time of cancer diagnosis. The AYAs suggested that the doctors could have informed parents about the risk and that the parents in turn could have given them the information when appropriate. Those who had received the information from their parents at a young age felt that they had “always” been aware of the risk of infertility, making it a natural part of their lives.

#### 3.1.2 Information satisfaction

The first information regarding the risk of infertility was provided by the pediatric oncologist or haematologist, but they did not delve deeper into the procedures for fertility preservation. Instead, procedural information was omitted at that point, and only later provided at the reproductive clinic. Most of the young individuals were satisfied with the information they received there. The information was detailed and conveyed both verbally and in writing, offering an understanding of the process. The written information allowed them to read and absorb the details at their own pace, which was appreciated. Beyond verbal and written information, the recommendation of websites and videos, along with explanations through drawings and visual aids, was seen as positive. The AYAs also sought others’ experiences online, considering it a valuable supplement, and felt content with the additional insights gained. However, they did this cautiously, as reading about others’ experiences made the impending treatment feel more real.

I actually became more nervous because then I thought: ‘Oh my God, I’m going to do this’. It was still okey [to read about others experiences] because then I found out what others had thought and how their process had been. So, that was comforting. But still, it became a bit more nerve-wracking because: ‘I’m also going to do that!’.

Female, age 18

The information about sperm cryopreservation was perceived as straightforward and clear. Despite being somewhat limited, the information was deemed sufficient. I contrast, information regarding oocyte cryopreservation was more extensive, and in some cases, individuals carefully selected among the available information to avoid feeling overwhelmed. Here, legal guardians played a role in helping to understand the information and provided support in managing emotions that arose.

Mum was present when we talked about everything so that I had a few more ears that could absorb all the information.

Female, age 20

#### 3.1.3 Insufficient information

Experiences of inadequate information were also described, particularly concerning the gynecological exams required for young females. There was a lack of a comprehensive overview of the process before their first visit to the fertility clinic, where the details of each procedure had not been outlined by their referring oncologists. Insufficient information, combined with limited prior experiences of gynecological examinations, led to confusion, a sense of being unprepared during the first meeting with the reproductive medicine specialist, and unmet expectations. This created a feeling of uncertainty among those undergoing the gynecological examination, which sometimes was challenging to cope with. Some AYAs described how the lack of information restricted their ability to communicate their feelings and thoughts during the examination. For some this resulted in the experience of the first gynecological examination as being the most challenging aspect of the entire process.

The first time was the worst. There were a lot of new impressions, and I really wasn’t prepared. If I had been prepared for it, it would have been much easier. [ … ] I didn’t expect it to be such a difficult moment.

Female, age 22

Since most of the young females were undergoing a gynecological examination for the first time, they felt that detailed information about the upcoming visit was essential. Specifically, they expressed a need for information regarding the gynecological examination and the vaginal ultrasound from their referring oncologists, believing that such details would aid in mental preparation and promote a sense of calm.

My experience, at least, is that if I had known exactly how it would look and what would happen once I got in there, it might have felt a bit easier.

Female, age 21

### 3.2 Freezing gametes - the process and healthcare encounters

#### 3.2.1 Preparatory examinations and treatment

Gynecological examinations and vaginal ultrasounds are a routine part of the process for cryopreservation of oocytes. However, as the information beforehand had not included this aspect, this was sometimes a surprise for the patients.

I didn’t even know that I was going to have a [vaginal] ultrasound. She said, ‘Should we do one?’ and I was like, ‘Uh, what?’ I panicked.

Female, age 18

For most young females, the feelings before gynecological examinations included nervousness, fear of pain, and a sense of being unprepared as they had not undergone such examinations before. Examinations were perceived as painful, uncomfortable, and demanding. Some wondered if the examination became difficult due to their inability to relax. With time, most individuals became accustomed to the procedure. They suggested that they would have been more comfortable if they had known more about this beforehand and also if the gynecologist had explained each step during the gynecological examination. They also reflected on if it would have been easier if it had been a female gynecologist.

Knowing that they needed to learn how to self-administer the hormone injections for ovarian stimulation sometimes caused negative feelings. Having support and assistance from a close relative made the situation feel manageable despite the discomfort that arose. However, even if the self-administration of the injections was challenging it also contributed to growing self-confidence and a sense of bravery.

Before, I was [ … ] very needle-phobic [ … ], it’s so difficult because you’re hurting yourself. It almost felt like I was stabbing myself with a knife. [ … ] But it was only the first time. And then, after that, when I saw that; ‘Okay, I didn’t die’ then it was easy to do it every night.

Female, age 22

#### 3.2.2 Oocyte retrieval and semen sample provision

The oocyte retrieval and semen provision were both perceived as frightening, yet exciting procedures. There was a sense of relief in getting it done and both females and males expressed a feeling of gratitude for the opportunity to undergo fertility preservation. However, the young women expressed more challenges surrounding the procedure than did the young men. Some individuals underwent egg retrieval multiple times, which increased the feeling of security. In addition, knowledge about the procedure made the entire process more manageable. Security and sense of comfort could also be established by having relatives for support. However, one individual who wanted to have a relative in the room during egg retrieval, were denied this due to Covid-19, which led to increased stress. Most of the young women experienced the local anesthesia as painful, and there were also descriptions of intense pain when the oocytes were retrieved. For some, the pain was manageable while for others, the pain was described as unbearable and inducing panic. This made it difficult to remain still during egg retrieval, and it was described as the most challenging aspect of FP.

I thought the [egg retrieval] was extremely painful. Much more painful than they had said it would be. It hurt so brutally. [The staff] said it would feel uncomfortable but should not hurt.

Female, age 21

For the young men, the semen sampling was uncomplicated and did not cause worries or problems. It was noted that the collection container used during the process was smaller than desired, making it cumbersome to handle. The men had all undergone fertility preservation in close connection with diagnosis and before starting their cancer treatment, the provision of the semen sample was therefore perceived as a relatively small part of the illness. The treatment was considered smooth and simple, with clear instructions and quick execution.

It was very clear. I received a letter about where to be, I went there, did what I had to do, and then I could go home.

Male, age 21

However, giving a semen sample was perceived as private and embarrassing, and those who were alone in the waiting room were thankful for it. Despite the uncomfortable situation, they were pleased to have completed the procedure.

Most young women and men in this study had chosen to communicate openly with their friends and family about undergoing fertility preservation measures, though without delving into specifics. Those who opted not to disclose this information did so because they perceived the time of being diagnosed with cancer as particularly challenging.

#### 3.2.3 Healthcare interactions throughout the process

Most of the young individuals felt that they received good treatment from the staff at the fertility clinic. They perceived the staff as attentive, caring, and responsive to their needs. The approach was tailored to their age and was undramatic. The staff was clear and available for questions, explaining and suggesting calming measures in a nervous and stressful situation.

Everyone there was really kind, nice, and very understanding because I was a bit emotional and nervous. They were calm and explained what they were doing.

Female, age 21

However, some experienced a difference in receiving care at the pediatric clinic compared to the fertility clinic. The feeling was that the staff at the fertility clinic had less time for each individual patient and less time to understand their needs and backgrounds. Communication gaps between the staff also occurred, causing fear and concern. A few young women experienced distress during the first gynecological examination.

My first gynecologist … but I understand that [he] might have found it difficult because I refused to relax, and I was really worked up…. he [said], ‘Okay, but we have to do this’ and I understood that. But I just wanted to leave and try another day.

Female, age 22

### 3.3 The decision to preserve gametes for one’s own sake

#### 3.3.1 The complexity of deciding to cryopreserve gametes

The decision to undergo fertility preservation was a straightforward and natural step for most participants. The decision was made quickly as it was perceived as a good opportunity and a smart choice to pursue. Despite the shock of receiving the news about the risk of infertility or a lack of knowledge about the fertility preservation procedure, there was no hesitation in making the decision.

I was still shocked about it I guess, but I had no doubt that it would be the best for me. And the option not to follow the advice wasn’t even considered.

Male, age 19

Not having to initiate the question of freezing gametes was perceived positively, and several of the young individuals were advised to undergo the procedure by their doctors. The message they received was that it was routine. If the doctor had not recommended fertility preservation measures, some of the young individuals probably would not have undergone the treatment, as the ability to have children in the future did not feel like a prioritized issue at that moment. A few revisited the fertility clinic repeatedly to discuss their options before making the decision. For some of the young individuals, discussing with a family member helped reach a decision about whether to undergo the treatment or not.

#### 3.3.2 Better safe than sorry

The decision to freeze gametes was considered a precautionary measure. Even though the risk of infertility, in some cases, was not particularly high, it felt safe and advantageous to take this step. The process of freezing gametes was not considered a problem but rather an insurance to enhance future possibilities of starting a family. By freezing gametes, the risk of regretting the decision not to freeze them disappeared, creating a sense of security. This decision was motivated by the desire to minimize worries and fostered joy in having a secure foundation.

They asked if I want to freeze or not. So, I just thought; ‘But why not just do it? Then you have it in case something happens’.

*Male, age 21*

Generally, participants expressed a low level of concern about their future fertility. The initial realization of the risk of infertility caused some to worry about the future. However, after undergoing fertility preservation, they placed their trust in these procedures and believed they would be able to start a family when they were older and ready.

[How does it feel to have your eggs in the freezer]? I think it’s kind of cool. [What are your thoughts about the future and the risk of infertility]? Well, I’ve been thinking like this; “Well, now there are eggs there, so if I can’t have children later, I can just go and get them.”

Women, age 18

## 4 Discussion

In this qualitative semi-structured interview study, we investigated the experience of fertility preservation in connection with cancer diagnosis at adolescent age among young AYAs after completion of cancer treatment. The study participants were generally not aware of the possible negative impact of cancer treatment on fertility prior to being informed through healthcare, and while the Swedish National Guidelines include a recommendation of fertility counselling at diagnosis (27), that information was not provided in a structured manner to all participants. This is in line with previous international studies indicating that the communication regarding late effects of cancer treatment is often poor (32, 33). Yet, a recommendation from the healthcare provider to proceed with FP has been shown to be among the most important factors when deciding to cryopreserve sperm (34, 35), and several study participants mentioned that the physician’s recommendation played an important part in their decision to proceed with FP. The main barriers to communication on fertility threats as sequelae of cancer treatment reported from oncologists include the lack of time, lack of knowledge, lack of specialized communication skills and the need to prioritize information to not overwhelm the patient (36). However, the study participants who received fertility-information at the same occasion in which they received their cancer diagnosis would have preferred to receive that information at a later appointment. The females who had been informed at a young age expressed least distress about the possibility of future infertility. Several individuals expressed a wish to have received information not only on fertility preservation options but also on the gynecological exams from their referring oncologists, prior to the first consultation at the fertility clinic.

Several AYAs in this study mentioned the role of their relatives in requesting and interpreting information, but also in decision making and as a support during examinations. This highlights how family and relatives can provide crucial support for young cancer patients. Previous studies have also underscored that the best way to protect fertility among underage patients is to actively include family in the discussion (37–39), but others note that the parents influence on decision making may be affected by their own perspective and predisposed by cultural beliefs, fear, or a wish for grandchildren (1, 40, 41). It has been shown that parents to female cancer patients are less likely to recommend FP to their children after being informed on the implications of the procedure (1). Parents have also been known to underestimate the importance future fertility in their children as their main focus is survival (39), thereby delaying discussions regarding future fertility. Often young age and a low level of patient autonomy increases the parental involvement (42, 43), but when the families take full control over the decision making it is rarely appreciated (37, 38, 44). Thus, the physician has a key role in keeping the patient’s perspective in focus, while thoroughly informing both patients and their families on the risk for late effects, and the risks and possibilities involved in counteracting them through FP. The PanCareLIFE Consortium and the International Late Effects of Childhood Cancer Guideline Harmonization Group (25) have developed recommendations on how to address treatment-induced infertility risk and FP in patients with childhood, adolescent, and young adult cancer. These include, among other things, that healthcare providers should foster patient autonomy by assessing emotional, psychological, and intellectual status during the informed consent process. Decisions about fertility preservation should prioritize the patient’s best interest, rather than the interests of parents, caregivers, or partners. Additionally, a two-stage consent process should be implemented: initially at diagnosis for harvesting and storing tissue, and later post-therapy at an appropriate age for deciding on the use of the stored material. An important aspect to improve equitable healthcare include also the provision of patient brochures and materials in languages spoken by minorities in specific countries, such as the reported efforts in Sweden to improve decision aids for FP of children and teenagers with cancer in that country (27).

The ethical considerations surrounding the choice to proceed with FP in female adolescents are considerable and include the respect of personal integrity, the consent process, the cultural aspects that can set transvaginal examinations in different contexts and the priority of fertility counselling when being challenged with a life-threatening disease (45). While female study participants were generally satisfied with the information provided at the fertility clinic regarding FP methodology and procedures needed, the first gynecological examination or transvaginal ultrasound was still distressing and some experienced pain and discomfort. The study participants expressed a wish for additional, and more complete, information regarding the vaginal exams from their referring physician, and also previous to, and during, the gynecological examination to feel safe. Cancer survivors may be affected by post-traumatic stress disorder or other forms of psychological distress (46, 47). This can intensify the strain on the individual, possibly contributing to a more challenging and painful experience during the vaginal exams compared to individuals without previous trauma. While it is unfortunately common for young women to experience some level of discomfort during their first gynecological examinations, studies indicate that clear communication surrounding the procedure and allowing for extra time during the appointment, where both the practitioner and the patient have time to establish a relationship and ask questions, can alleviate fear and also reduce anxiety and pain (48–50). Similar recommendations are also given on how to handle previous trauma in gynecological settings (51, 52). The young women also expressed that they would have felt more comfortable with a female gynecologist. This has been observed in multiple studies, where younger women have a particularly strong preference for a female practitioner (49, 53).

The young male participants generally had a positive experience of semen banking They received limited information regarding the methodology, but felt it was sufficient. Discomfort was mostly centered around a feeling of embarrassment, but all participants considered the procedure uncomplicated and as something they were pleased to have completed. The differences between the male and female experience of the procedures required to cryopreserve gametes underscores the gender-based inequality also observed in previous studies of fertility preservation information and access in the context of cancer (19).

The study participants showed less verbalized concerns about future fertility than noted in similar studies on an older population (54, 55), perhaps because family building is still in the distant future with the mean age of AYAs in this cohort being 19,4 years, while the mean age for a first child in Stockholm is 31,2 years (56). The decrease in verbalized concerns may also indicate a shift in the perception of possible infertility. There is a possibility that young adults no longer perceive the same stigma surrounding the ability to have biological children as previous generations did. The participants’ lack of concern and view of FP as a “back-up” for the future might also reflect on either an inability of the healthcare provider to adequately convey the uncertainty of future ART, or improved means of discussing the possibility of future infertility. Nevertheless, having undergone FP seems to have provided a sense of security regarding future fertility and our results indicate that undergoing FP has buffered fertility distress.

A strength of this qualitative study is that we are able to highlight the experiences of an often-overlooked age group, where the study participants all underwent FP in their late teens. This work correlates with quantitative data addressing the same topic but has enabled a deeper insight and allowed the very young FP patients to give voice to their experience of FP.

There are, however, limitations to a qualitative approach such as not being able to control the narrative, the risk of participants avoiding sensitive topics and the risk of not reaching saturation. There was a risk of responder bias since not all patients who were contacted agreed to interview. This may have excluded patients less inclined to reminisce further about their treatment experiences and involving them may have given more contrasting views. Also, all study participants had successfully preserved gametes which may influence their experience positively. Finally, this study was conducted at a single academic center in an optimum resource setting (57), and it is difficult to know whether similar findings would be gained at other hospitals acting under the same guidelines. Different criteria for FP access and funding, as well as different pathways of referral, are likely to influence the FP experience. Also, a low resource center might not have access to all FP options, might not have national standards for eg. FP counselling, nor for offering the same options for future fertility treatments (57).

In conclusion, a first gynecological examination at a young age, combined with a recent cancer diagnosis, presents unique challenges that need to be further acknowledged by the healthcare providers. The situation requires special considerations and tailored guidelines at reproductive health units. It is essential to systematically address the patients’ needs to provide appropriate care and support for this group. In summary, based on our study results, we recommend: 1) Fertility information well in advance of the FP decision-making, first provided by the pediatric oncologist. 2) Information on the planned gynecological procedures to be provided before and during the examinations, explaining what is going to happen, and the objective of the exam. 3) Time and encouragement to ask questions. 4) When possible, a same sex practitioner experienced in trauma informed care to conduct the first pelvic exams.

The documented experience of undergoing fertility preservation as a teenager highlights gaps in communication about fertility risks and treatment options. Healthcare provider recommendations significantly influence decisions to pursue fertility preservation, while family support plays a crucial role both in decision making as well as a support during treatment. Improved communication strategies tailored to both the patients and their families are essential for informed decision-making and supportive care. Gender disparities exist, both regarding information and experience of the treatment. While young the young men were generally satisfied, the young women expressed discomfort and emphasized a preference for female gynecologists and the importance of early and clear communication. Specifically, improved communication at referral and more information regarding the vaginal examinations prior to, and during, the exams were requested. Possible improvements include the training of oncologists on the specific needs of AYAs with cancer to gain this knowledge, thus facilitating patient referrals and future gynecological consultations, or the availability of a reproductive specialist consultation at the oncologic center. Independent of their treatment experience, undergoing fertility preservation indicated by a cancer diagnosis at young age appears to provide a sense of security regarding future fertility and mitigates fertility-related distress in both male and female teenage patients, who appreciated the opportunity to access fertility preservation options.

## Data Availability

The datasets presented in this article are not readily available because any data shared could contribute to identify the participants. Requests to access the datasets should be directed to kenny.rodriguez-wallberg@ki.se.
